# The Enteric Nervous System and Its Emerging Role as a Therapeutic Target

**DOI:** 10.1155/2020/8024171

**Published:** 2020-09-08

**Authors:** Mark A. Fleming, Lubaina Ehsan, Sean R. Moore, Daniel E. Levin

**Affiliations:** ^1^Department of Surgery, Division of Pediatric Surgery, University of Virginia School of Medicine, Charlottesville, VA 22908, USA; ^2^Department of Pediatrics, Division of Pediatric Gastroenterology, Hepatology, and Nutrition, University of Virginia School of Medicine, Charlottesville, VA 22908, USA

## Abstract

The gastrointestinal (GI) tract is innervated by the enteric nervous system (ENS), an extensive neuronal network that traverses along its walls. Due to local reflex circuits, the ENS is capable of functioning with and without input from the central nervous system. The functions of the ENS range from the propulsion of food to nutrient handling, blood flow regulation, and immunological defense. Records of it first being studied emerged in the early 19^th^ century when the submucosal and myenteric plexuses were discovered. This was followed by extensive research and further delineation of its development, anatomy, and function during the next two centuries. The morbidity and mortality associated with the underdevelopment, infection, or inflammation of the ENS highlight its importance and the need for us to completely understand its normal function. This review will provide a general overview of the ENS to date and connect specific GI diseases including short bowel syndrome with neuronal pathophysiology and current therapies. Exciting opportunities in which the ENS could be used as a therapeutic target for common GI diseases will also be highlighted, as the further unlocking of such mechanisms could open the door to more therapy-related advances and ultimately change our treatment approach.

## 1. Introduction

The gastrointestinal (GI) tract is innervated by an extensive intrinsic network of ganglion-rich nerve connections known as the enteric nervous system (ENS) [1, 2]. The human ENS contains approximately 400-600 million neurons that can be found in two major networks—the myenteric and submucosal plexuses, which are also known as *Auerbach's* and *Meissner's* plexus, respectively [3–5]. The ENS is the largest and most complex unit of the peripheral nervous system and is located within the walls of the GI tract, extending from the esophagus to the anal canal [6, 7]. In fact, it has been classified as the third division of the autonomic nervous system in addition to the sympathetic and parasympathetic divisions by Langley during the early 20^th^ century [1, 2, 7, 8]. The submucosal plexus lies just beneath the mucosal layer of the gut and is predominantly found in the small and large intestines, whereas the myenteric plexus is found between the circular and longitudinal layers of smooth muscle and can be found along the entire length of the GI tract [1, 6, 9]. Although it receives central nervous system (CNS) input via the vagus nerve and thoracolumbar and lumbosacral spinal cord, it has been shown very early on to act independently of the CNS [3, 10, 11]. The ENS possesses peristaltic motor, secretory, and immunological function in addition to more complex behaviors such as nonpropulsive mixing or segmentation, slow orthograde propulsion via the migrating myoelectric complex (MMC), retropulsion of noxious substances, and modification of nutrient handling and changing of local blood flow [1, 4]. This system is supported by peripheral glial cells called enteric glia that helps the ENS maintain the integrity of the epithelial barrier and that have been shown to play a role in intestinal inflammation and interaction with the microbiome [4, 12, 13]. In this review [14], we will provide a brief general overview of the history, embryology, anatomy, and function of the ENS to date as it relates to the small intestine in a way that the average reader can understand. We hope to make a novel contribution to the literature by connecting common GI disorders with specific neuronal pathophysiology and therapies and also summarize opportunities for future investigation including the potential role of the ENS and the intestinotrophic effect of glucagon-like peptide 2.

## 2. History

The study of the ENS dates back to the nineteenth century when German anatomist and neuropathologist, Leopold Auerbach, was credited with the discovery of *Plexus myentericus Auerbachi* or Auerbach's (myenteric) plexus in the mid-19^th^ century [15, 16]. This was followed by the discovery of Meissner's (submucosal) plexus by German anatomist and physiologist, Georg Meissner, around the same time [16]. In 1899, two English scientists, Bayliss and Starling, published a series of articles detailing their experiments on the function of these plexuses and subsequently described the “Law of the Intestine” [3, 17]. This was the first demonstration of the peristaltic reflex and the ENS' ability to function independently of the CNS. This law was reproduced and further characterized by other early pioneers in the field of neurogastroenterology [1, 10, 18]. More specifically, Trendelenburg was the first to reliably reproduce the peristaltic reflex in a completely isolated intestine of the guinea pig with a stimulus that was easily adjustable [10]. The first attempts of morphological classification were made by Cajal and Dogiel who studied their morphology and microarchitecture identified by silver impregnation methods [15, 19]. In the 20^th^ century, multiple attempts at further classification in an effort to support or refute Dogiel's efforts were made [6, 19, 20].

## 3. Embryology

**Figure 1:**
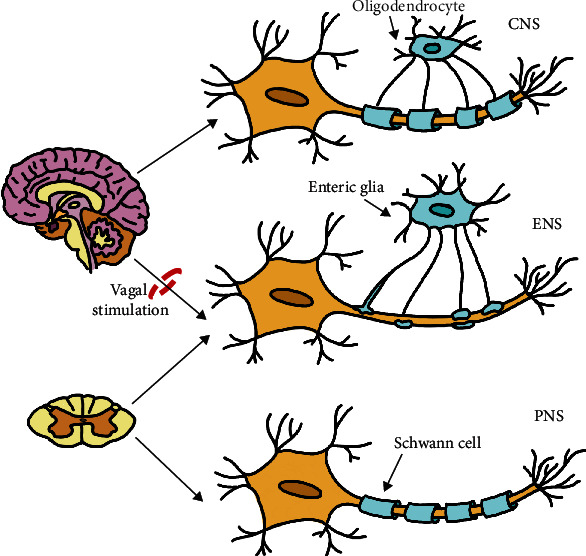
Similarities and differences of the ENS, CNS, and PNS and their supportive cells. CNS: central nervous system; ENS: enteric nervous system; PNS: peripheral nervous system.

The development of the ENS has largely been studied in murine and avian embryo models [21–23]. The majority of progenitor cells have been shown to originate and migrate from the vagal level of the neural crest along defined pathways ahead of the descending vagus nerve fibers, picking up cues from the microenvironment along the way before differentiating within the wall of the GI tract [22–25]. They migrate as chains proximodistally within the outer gut mesenchyme and remain in contact with one another for directional migration [26, 27]. A large subset of the vagal enteric neural crest-derived cells take a shortcut through the dorsal mesentery from the ileum to a loop of postcecal bowel, presumably the ascending colon [26]. Additionally, a small group of sacral crest-derived cells migrate to the bowel through the somatic mesenchyme and enter it with the extrinsic sacral nerves giving rise to approximately 20% of postumbilical neurons [21, 28–30]. However, this has recently become controversial as a recent report has called for the redefining of the sacral innervation [31]. During human gestation, the ENS becomes functional during the last trimester and continues to develop following birth [11]. Given the complexity of its development with the migration of cells from the neural crest, the ENS has many unique organizational features that make it similar to the CNS [32]. It lacks much of the internal collagen that creates connective tissue between neurons, and the supportive cells—enteric glia—resemble the astroglia of the CNS and less so Schwann cells (Figure 1) [12, 33–36]. In this regard, the symptoms of obstruction seen in Hirschsprung's disease (Table 1) occurs when a segment of bowel is deprived of ganglion cells secondary to defective migration of enteric glia. This highlights the importance of the ENS to the gut and its motor function [37].

## 4. Anatomy and Function

**Figure 2:**
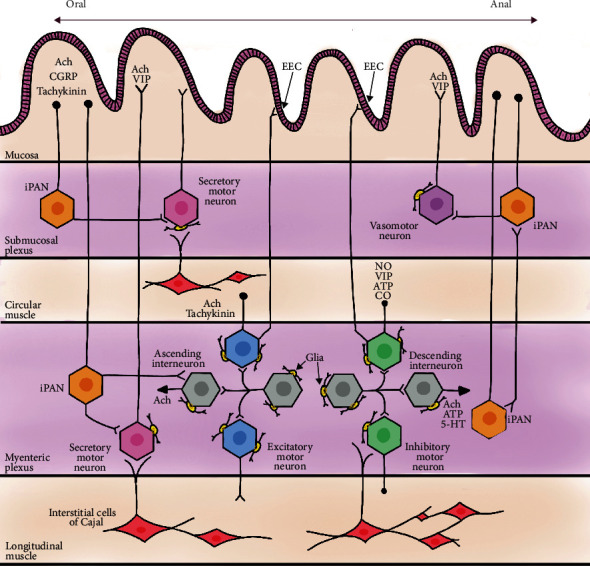
Drawing of the layers of the small intestine showing the complex ENS network, differentiated cells, and neurotransmitters. iPAN: intrinsic primary afferent neuron; EEC: enteroendocrine cell; Ach: acetylcholine; CGRP: calcitonin gene-related peptide; VIP: vasoactive intestinal peptide; ATP: adenosine triphosphate; NO: nitric oxide; CO: carbon monoxide; 5-HT: serotonin.

The ENS consists of up to 20 different types of neurons, containing more than all the sympathetic and parasympathetic ganglia combined and a similar amount of neurons to what is in the spinal cord [11, 33]. The major categories as observed by Furness et al. in the Burnstock laboratory include intrinsic primary afferent neurons (IPANs), motor neurons, and interneurons (Figure 2) [1, 9, 11, 15, 38]. These neurons are further classified based on their morphological (Dogiel types I-VII), electrical (types S and AH), chemical (neurotransmitters), and functional properties [15, 20, 39].

### 4.1. Intrinsic Primary Afferent Neurons

The intrinsic primary afferent neurons (IPANs) are some of the first sensory neurons to detect the physical state of the intestine. They are located in the submucosal and myenteric plexuses [40]. The primary neurotransmitters of IPANs are acetylcholine, calcitonin gene-related peptide (CGRP), and tachykinin; the secondary neurotransmitter is undetermined [4, 9, 41]. Morphologically, IPANs are classified as Dogiel type II [40]. They are round or oval in shape and create multiaxonal or pseudounipolar synapses with multiple types of neuronal elements to form intrinsic reflex circuits [40, 42, 43]. In the guinea pig model, myenteric sensory neurons of Dogiel type II morphology make up the majority (~97%) of neurons that project to the mucosa [44]. In the more complicated porcine model, most of the mucosal-projecting neurons live in the submucosal plexus, and a minority (12%) are located in the myenteric plexus. Of the latter group, approximately 23% of the myenteric neurons projecting to the mucosa are Dogiel type II, highlighting a stark difference in the proportion of primary afferent myenteric neurons between both models [20]. Notably, guinea pig and murine models show IPANs to be responsive to mucosal mechanical distortion, to distortion of their processes in the external muscle layers, and to chemicals that interact with the mucosa [40, 45–48]. Within this context, primary afferent nerves such as IPANs have been investigated with respect to their altered excitability and influence on motor activity in inflammatory disorders of the gut (Crohn's, ulcerative colitis, or infectious), and innovative therapeutic targets have been identified (Table 1) [49–51].

### 4.2. Motor Neurons

Motor neurons of the ENS innervate the circular and longitudinal muscle layers, intrinsic arterioles, and epithelium including enteroendocrine cells [9, 11, 32]. Five broad types have been identified as excitatory, inhibitory, secretomotor, vasomotor, and neurons innervating enteroendocrine cells [9]. The excitatory motor neurons predominantly use acetylcholine as their neurotransmitter with a small component of tachykinins (substance P) [9, 41, 80, 81]. They mainly innervate the circular muscle extending near the boundary of the submucosa and also project more orally compared to the inhibitory neurons [47]. The inhibitory motor neurons primarily use nitric oxide as their neurotransmitter, with vasoactive intestinal peptide (VIP), adenosine triphosphate (ATP), and carbon monoxide (CO) as secondary ones [82–85]. They project to muscle that is close (within 2 mm) to their cell bodies in the anal direction [47]. The excitatory motor neurons stimulate smooth muscle contraction whereas the inhibitory neurons discharge in a continuous fashion, and so inactivity of inhibitory neurons results in propulsive contraction towards the anus [15, 16]. The effects of both excitatory and inhibitory motor neurons have been shown in part to be mediated by the interstitial cells of Cajal (ICC), and this concept is supported by the presence of NO and excitatory tachykinin transmitter receptors on these cells [47, 86–90]. Abnormal excitatory and inhibitory input due to the effect of the autonomic nervous system (sympathetic inhibition of Ach release), neurotransmitters (VIP, NO, substance P, and CGRP), hormones such as corticotropin-releasing factor (CRF), endogenous opioids, and bowel manipulation has been shown to result in various forms of gastrointestinal dysmotility in animal experiments [70, 76, 91]. In addition, the inhibitory effect of anesthetics and morphine on gastrointestinal motility has been demonstrated in humans [76]. These identified mechanisms support the idea that the cause of intestinal pseudoobstruction and postoperative ileus is likely multifactorial and that the targeting of these pathways could lead to preventative and/or curative therapies (Table 1).

Secretomotor neuron cell bodies are located in the submucosal and myenteric plexuses; however, they are a part of secretomotor circuits that involve IPANs with nerve endings in the mucosa [47]. Their activity is initiated through the interaction of luminal contents such as glucose with the mucosa or by toxins such as cholera and enterotoxins [15, 92, 93]. Secretomotor neuron main function is to secrete chloride ions into the intestinal lumen dragging water molecules with them. They consist of a cholinergic and a noncholinergic type [47]. The noncholinergic type uses VIP or a related peptide as its primary neurotransmitter and mediates most of the local reflex response in contrast to the cholinergic neurons that act on muscarinic receptors on the mucosal epithelium [15, 93, 94].

Similar to the secretomotor neurons, the vasomotor neuron cell bodies are located in the submucosal plexus ganglia and their activity is presumed to also be mediated by IPANs, though not significantly [15, 47, 95]. They are the least studied type of motor neuron; however, there is enough evidence to suggest that they are split into cholinergic and noncholinergic neurons, with acetylcholine as the likely primary neurotransmitter and VIP as secondary [47, 95–98]. It is easy to understand how the secretomotor and vasomotor neurons work in tandem to regulate epithelial secretion and blood flow, and it is important to note that these reflexes are under extrinsic modulation via the sympathetics [9].

Enteroendocrine cells are highly specialized cells that reside in the intestinal mucosa interacting with various chemical and mechanical stimuli within the gut's lumen [15]. The major transmitters include, but are not limited to, cholecystokinin (CCK), secretin, somatostatin, serotonin (5-HT), corticotrophin-releasing factor, gastrin, leptin, ghrelin, and glucagon-like peptide 2 (GLP-2) [11, 15, 99]. They are released from these cells and interact with afferent nerve fibers in the lamina propria which in turn communicate with excitatory and inhibitory motor neurons [15]. The production of GLP-2 by enteroendocrine cells is worth highlighting further for the purpose of this review. There is evidence to suggest that these cells detect and participate in the transport of glucose across the mucosa via the activation of glucose transporters by GLP-2 [100]. However, the receptor for GLP-2 is on submucosal neurons as well, which implies that glucose transport could also be mediated by enteric neurons that are excited by GLP-2 [101, 102].

### 4.3. Interneurons

There are two main types of interneurons—ascending or orally directed interneurons and descending or anally directed interneurons [19]. They are primarily located in the myenteric plexus. Just like the motor neurons, the interneurons' primary neurotransmitter is acetylcholine. Furthermore, ATP has been identified as a secondary neurotransmitter especially in the descending type [47, 103, 104]. However, there is conflicting evidence on whether or not 5-HT is also a secondary neurotransmitter of the descending interneuron [40, 47, 104]. In the guinea pig, one type of ascending and three types of descending interneurons have been identified and have been noted to form chains that extend the length of the GI tract [9, 47]. The majority of the input to the ascending interneurons comes from IPANs, and the remaining input is from other ascending interneurons [47]. In contrast, the descending interneurons receive very little input from IPANs but rather from other descending interneurons. It is therefore thought that the descending interneurons are heavily involved in the MMC of the small intestine [40, 47].

## 5. Supporting Cells

### 5.1. Enteric Glia

The enteric glia are the supporting, nonneuronal cells of the myenteric and submucosal plexuses with an approximate ratio of glia to neurons of 2-3 to 1 [36, 79]. They are believed to originate from the neural crest and migrate to the bowel either at the time that the vagal- and sacral crest-derived cells do or later during gut development at the time of extrinsic nerve migration [12, 13]. A unique characteristic is the abundance of glial fibrillary acidic protein (GFAP) that is present in their cytoplasm compared to Schwann cells [12]. This is a result of the large amount of 10 nm intermediate filaments known as “gliofilaments” that they possess [34, 105, 106]. The enteric glia are far more irregular in shape compared to Schwann cells, and they have long processes that radiate out and terminate into small swellings called “end feet” forming an incomplete glial sheath that partially separates the myenteric neurons from the surrounding connective tissue [36]. Several neurotransmitters such as acetylcholine, catecholamines, glutamate, adenosine, and serotonin activate enteric glia [13]. They nourish neurons, maintain homeostasis, and are now being increasingly acknowledged as active regulators of multiple physiological processes [13, 79]. There is some evidence to suggest that enteric glia may have a neurosecretory function, just like astroglia are believed to play a role in controlling ionic flux in the CNS [36]. Enteric glia interact with various other nonneuronal cell types such as enterocytes, enteroendocrine, and immune cells which speak to their emerging role in regulating various intestinal functions and their involvement in pathological disorders such as diarrhea, Parkinson's disease, and Creutzfeldt-Jakob disease (Table 1) [13, 79].

### 5.2. Interstitial Cells of Cajal

The interstitial cells of Cajal (ICC) have been called the pacemakers of the GI tract due to their ability to produce cyclic spontaneous depolarization and slow waves described as the basic electrical rhythm [107]. They are responsible for initiating slow waves within the GI tract smooth muscle layers due to the lack of unique ion mechanisms within smooth muscle cells necessary to independently produce them [108]. Slow waves are needed to depolarize smooth muscle cells enough to activate calcium influx and trigger excitation-contraction coupling [109]. Furthermore, studies in humans and mice have suggested a mechanosensitive function induced by muscle stretch that then influences slow wave frequency and smooth muscle chronotropy; however, the underlying mechanisms are not fully understood [110, 111]. Experiments in avian and murine models have shown that ICC are derived from mesenchymal cells induced by kit signaling and develop independently from the enteric neuron [53, 112, 113]. Thus, they express c-kit—the marker by which these cells are identified—and a transmembrane receptor that induces receptor tyrosine kinase activity after the binding of its ligand, steel factor (kit ligand or stem cell factor) [107]. As mentioned earlier, they also possess receptors for tachykinins and NO produced by excitatory and inhibitory neurons, respectively, as well as for 5-HT [114, 115]. They are characterized by an elongated, fusiform body with few processes and are located at the junction of motor neurons and smooth muscle cells, forming connections similar to traditional synapses [107–109]. Moreover, ICC are involved in an integrated functional syncytium comprised of smooth muscle cells, ICC, and platelet-derived growth factor-positive cells (i.e., SIP syncytium) [109, 116]. From a pathological perspective, loss of ICC has been observed in a variety of human intestinal motility disorders including chronic intestinal pseudoobstruction (CIPO), Hirschsprung's disease, inflammatory bowel diseases (IBD), mechanical obstruction, and slow transit constipation (Table 1) [53, 109, 117, 118]. Though this remains controversial, ICC have also been suggested as a source of gastrointestinal stromal tumors (GIST) and as one of the reasons for the effectiveness of tyrosine kinase inhibitor, imatinib [53, 119].

## 6. Current ENS-Targeted Therapies of Common GI Diseases

In addition to the examples summarized in Table 1, several common GI diseases have established ENS-targeted therapies. A number of these therapies will be discussed below in more detail and within the context of the disease.

### 6.1. Achalasia

Botulinum toxin A is a highly selective neurotoxin that inhibits acetylcholine release from nerve terminals including those of the enteric neurons [120]. It exerts its effects by first gaining entry into the neuron via synaptic vesicle (SV2) receptors [121]. The toxin is produced by the bacterium *Clostridium botulinum* and was first isolated in the 1940s [121]. Since then, it has been extensively studied and is now used to treat a range of disorders affecting the nervous, urologic, ophthalmologic, dental, and gastrointestinal systems, among others [121, 122]. Achalasia is a rare motility disorder that affects the lower esophageal sphincter—resulting in dysphagia, chest pain, food intolerance, and recurrent aspirations that cause pneumonia [120, 123]. Although the root cause remains elusive, we now know that it is due to the loss of inhibitory neurons of the myenteric plexus resulting in failure of the lower esophageal sphincter (LES) to relax [124, 125]. While nitrates and calcium-channel blockers are often used to pharmacologically treat achalasia by acting on smooth muscle, botulinum toxin A has emerged as a safe and effective treatment that targets the excitatory motor neurons of the ENS [120, 126].

### 6.2. Slow Transit Constipation

Serotonin type 4 (5-HT4) receptor agonists such as prucalopride, tegaserod, cisapride, velusetrag, and naronapride have been shown to improve colonic motility in patients who suffer from slow transit constipation (STC) [127, 128]. Besides constipation, other symptoms of STC include abdominal pain, nausea, vomiting, and distention [129]. It is characterized by persistent constipation secondary to slow colonic transit that does not respond readily to dietary changes or laxatives [130]. Although the etiology is not entirely clear, this colonic dysmotility has been linked to a disruption of the autonomic and enteric nervous systems, as well as the neuroendocrine system [130–132]. More specifically, a reduced number of myenteric plexus neurons and ICC cells have been demonstrated in patients with slow transit constipation [125, 131–133]. Prokinetic 5-HT4 receptor agonists exert their effect by binding to the 5-HT4 receptor on the enteric neuron leading to the release of acetylcholine and other mediators of excitatory pathways that increase motility [134]. Of note, the nonselective 5-HT4 agonists—tegaserod and cisapride—have fallen out of favor due to their adverse cardiac side effects; however, the more recent and highly selective agonists such as prucalopride, velusetrag, and naronapride have been shown to be safer and much more tolerated [127]. Interestingly, prucalopride has also been shown to have a neuroprotective effect on the enteric nervous system [134].

### 6.3. Gastroparesis

As is the case in other gastrointestinal dysmotility disorders, 5-HT4 receptor agonists like prucalopride have also proven to be effective in the treatment of gastroparesis [135]. Symptoms of gastroparesis include early satiety, nausea, vomiting, postprandial fullness, and distention which are a result of delayed gastric emptying in the absence of a true mechanical obstruction [136]. The most common etiologies are idiopathy, diabetes, and postsurgery [137]. Both vagal and ENS dysfunctions have been demonstrated in humans with gastroparesis where the loss and injury of ICC and abnormal inhibitory and excitatory motor neurons, as well as a decrease in the number of enteric neurons, all contribute to its pathogenesis [135, 138–140]. Metoclopramide, primarily a D2 receptor antagonist with some 5-HT4 receptor agonism, leads to a gastric prokinetic effect by antagonizing synaptic D2 receptors and stimulating 5-HT4 receptors on the enteric neuron [141, 142]. Due to its ability to cross the blood-brain barrier, it also exerts a central antiemetic effect; however, it can cause extrapyramidal side effects as well which is why its use is limited to 12 weeks [135, 142]. Other pharmacologic treatments of gastroparesis symptoms that act on the enteric nervous system via their respective receptors include domperidone, levosulpiride, erythromycin, motilin, ghrelin, and ondansetron [125, 136, 142–144]. Of note, the approval and use of these medications vary from country to country.

### 6.4. Irritable Bowel Syndrome

Another class of drugs, not previously discussed, act on the opioid receptors of the ENS (e.g., Eluxadoline and Loperamide) [145–147]. For example, they target the ENS to provide relief of gastrointestinal (primarily diarrheal) symptoms seen in irritable bowel syndrome (IBS) patients [148]. Similarly, 5-HT3 receptor antagonists (e.g., alosetron and ondansetron) have also proven to be of clinical benefit in controlling diarrhea-predominant IBS [149, 150]. IBS is typically diagnosed using the Rome IV criteria—a 3-month history of recurrent abdominal pain for at least 1 day per week in addition to experiencing two or more symptoms such as defecation, change in stool frequency, or change in stool form [148, 151]. It is classified into 4 subtypes (IBS-diarrhea, IBS-constipation, IBS-mixed, and IBS-unsubtyped), but therapies targeting the ENS primarily treat the IBS-diarrhea subtype [152]. Pathogenesis as it pertains to the ENS is due to alterations in sensory and motor function; however, overall, it is not well-understood [148, 153]. Loperamide acts via the mu-opioid receptors on the enteric neuron to slow intestinal transit [152]. The newer therapy of the two, Eluxadoline, acts via the gamma-, kappa-, and mu-opioid receptors to exert its antidiarrheal effects in IBS-diarrhea patients and is currently being investigated in a randomized clinical trial for the treatment of diabetic diarrhea (ClinicalTrials.gov, NCT04313088) [145, 147].

## 7. GLP-2 and Short Bowel Syndrome

Glucagon-like peptide 2 (GLP-2) is heavily involved in the digestive process and is cosecreted, along with its sister hormone GLP-1, from enteroendocrine L-cells of the small and large intestines [68, 154]. Studies have demonstrated its ability to inhibit gastric emptying and gastric acid secretion stimulated by meals, as well as its role in increasing intestinal barrier function as part of the immune response [68, 155, 156]. Glucagon-like peptide 2 also regulates many intestinal adaptive processes including epithelial proliferation, apoptosis, and inflammation [68, 157]. To exert its effects, GLP-2 interacts with its receptor on the enteric neurons, subepithelial myofibroblasts, and intestinal endocrine cells as demonstrated in the mouse, rat, pig, and human intestines [66, 68, 158–160]. The GLP-2 receptor is highly selective for its cognate ligand, GLP-2, and does not allow effective binding of its structurally related peptide, GLP-1 [68, 159]. When the enteric neuron is exposed to GLP-2, it results in expansion of the mucosal epithelium of the small and large intestines and exerts antiapoptotic actions in the normal and injured intestine by inducing the expression of cell survival genes and proteins [64, 65, 68, 161]. Clinically, this has benefited both adult and pediatric patients who are suffering from short bowel syndrome (SBS) through the development of the GLP-2 analog, Teduglutide (Table 1) [64, 65, 69, 162, 163]. Therefore, the clinical success of Teduglutide makes sense as GLP-2 was previously linked to the regulation of nutrient absorption in several models, as well as in healthy human subjects [164–167]. Furthermore, GLP-2 has been shown to selectively increase visceral blood flow in pigs and healthy humans, as well as in SBS patients [168–170]. In the study of SBS patients, the increase in blood flow correlated with the length of their remaining intestine, implying that GLP-2 exerted metabolic effects on the intestine itself as opposed to the vasculature [170]. In fact, this was demonstrated earlier in a representative porcine model (given its similarity to humans) where GLP-2-induced stimulation of visceral blood flow was mediated by intestinal endocrine cells and the enteric neuron, reenforcing its clinical role in the treatment of SBS patients and making it a potential therapeutic target for low-flow gut diseases such as nonocclusive mesenteric ischemia [66]. Thus, the unlocking of these GLP-2 mechanisms has opened the door to a broad avenue of research looking at the role of GLP-2, the enteric neuron, and the repair, improvement, and maintenance of mucosal integrity and nutrient absorption.

## 8. Conclusion

The enteric nervous system is the largest and most complex unit of the peripheral nervous system, with ~600 million neurons releasing a multitude of neurotransmitters to facilitate the motor, sensory, absorptive, and secretory functions of the gastrointestinal tract. The enteric nervous system receives regulatory signals from the central nervous system via vagal, thoracolumbar, and lumbosacral input; however, it is also capable of independent function as evidenced by the intestinal peristaltic reflex. The involvement of the enteric nervous system in pathological disorders of the gastrointestinal tract, and the presence of receptors on the enteric neuron for enteric hormones and its transmitters, provides the foundation for current and future targeted therapies that could help patients suffering from a broad range of gastrointestinal disorders.

## Figures and Tables

**Table 1:** Additional examples of ENS involvement in various GI diseases and its role as a therapeutic target.

Disorder	ENS involvement^∗^	Clinical feature(s)	Therapeutic targets^†^
Gut inflammation	Proinflammatory cytokine-mediated alteration of afferent nerves and enteric glia [49, 50]	Specific to inflammatory disorder (Crohn's, ulcerative colitis, or infectious diarrhea)	IL-1*β*, TNF-*α*, mast cell products, 5-HT_3_ agonists, substance P, and CGRP [49]
Hirschsprung's disease	Aganglionosis of myenteric and submucosal plexuses due to defective migration of neural crest cells, disruption of ICC network [52, 53]	Chronic constipation, obstruction, failure to thrive, toxic megacolon [37]	Neuronal stem cell therapy; exploitation of proliferative ICC signaling pathways [53–56]
Infectious secretory diarrhea	Prostanoid- and 5HT-mediated stimulation of secretomotor neurons triggered by inflammatory mediators released by mast cells and neutrophils [57–59]	Loose and watery stools, +/- blood, abdominal pain, dehydration, nutrient loss, sepsis [60]	Neural blockade, Loperamide [57–59, 61]
Diabetic diarrhea	Diabetic autonomic neuropathy resulting in vagal and sympathetic nerve damage [62]	Nocturnal watery and painless stools, +/- incontinence [63]	Unclear, codeine phosphate [63] Eluxadoline (NCT04313088)
Short bowel syndrome	Intestinotrophic effects mediated by presence of GLP-2 receptor on submucosal neurons and endocrine cells [64–66]	Intestinal failure resulting in malabsorption and malnutrition [67]	GLP-2 analogs such as Teduglutide [65, 67–69]
Chronic intestinal pseudoobstruction (CIPO)	Hyperactive but disorganized excitatory motor neurons due to dysfunctional or damaged inhibitory motor neurons and loss of ICC [53, 70–72]	Nausea, vomiting, abdominal pain, distention, constipation, diarrhea, malnutrition [71, 72]	Metoclopramide, erythromycin, octreotide, and neostigmine; proliferative ICC pathways [53, 72, 73]
Postoperative ileus	Increased sympathetic activity resulting from inhibitory neural reflexes from the spinal cord; release of inhibitory neurotransmitters and ICC loss (NO, VIP, substance P) [74–76]	Nausea, vomiting, abdominal distention, obstipation [77]	Octreotide and CGRP as potential therapies; proliferative ICC pathways [53, 73, 75, 76]
Parkinson's disease (PD) and Creutzfeldt-Jakob disease	Deposits of alpha-synuclein and misfolded proteins found in enteric neurons/glia [78, 79]	GI dysfunction, constipation, reservoir of prions [79]	Explore ENS role as a biomarker in these diseases

^∗^May not represent a singular pathophysiological process of the disease. ^†^Therapeutic targets may or may not be approved for clinical use.
